# Impact of nighttime procedures on outcomes after liver transplantation

**DOI:** 10.1371/journal.pone.0220124

**Published:** 2019-07-22

**Authors:** Felix Becker, Thekla Voß, Annika Mohr, Anne-Sophie Mehdorn, Katharina Schütte-Nütgen, Stefan Reuter, Iyad Kabar, Eike Bormann, Thorsten Vowinkel, Daniel Palmes, Jens G. Brockmann, Andreas Pascher, Ralf Bahde, Linus Kebschull, Thomas Vogel

**Affiliations:** 1 Department of General, Visceral and Transplant Surgery, University Hospital Münster, Münster, Germany; 2 Department of Internal Medicine D, Division of General Internal Medicine, Nephrology and Rheumatology, University Hospital Münster, Münster, Germany; 3 Department of Internal Medicine B, Gastroenterology and Hepatology, University Hospital Münster, Münster, Germany; 4 Institute of Biostatistics and Clinical Research, University Hospital Münster, Münster, Germany; Medizinische Universitat Graz, AUSTRIA

## Abstract

**Background:**

Sleep deprivation is a well-known risk factor for the performance of medical professionals. Solid organ transplantation (especially orthotopic liver transplantation (oLT)) appears to be vulnerable since it combines technically challenging operative procedures with an often unpredictable start time, frequently during the night. Aim of this study was to analyze whether night time oLT has an impact on one-year graft and patient survival.

**Material and methods:**

Deceased donor oLTs between 2006 and 2017 were retrospectively analyzed and stratified for recipients with a start time at day (8 a.m. and 6 p.m.) or at night (6 p.m. to 8 a.m.). We examined donor as well as recipient demographics and primary outcome measure was one-year patient and graft survival.

**Results:**

350 oLTs were conducted in the study period, 154 (44%) during daytime and 196 (56%) during nighttime. Donor and recipient variables were comparable. One-year patient survival (daytime 75.3% vs nighttime 76.5%, *p* = 0.85) as well as graft survival (daytime 69.5% vs nighttime 73.5%, *p* = 0.46) were similar between the two groups. Frequencies of reoperation (daytime 53.2% vs nighttime 55.1%, *p* = 0.74) were also not significantly different.

**Conclusion:**

Our retrospective single center data derived from a German transplant center within the Eurotransplant region provides evidence that oLT is a safe procedure irrespective of the starting time. Our data demonstrate that compared to daytime surgery nighttime liver transplantation is not associated with a greater risk of surgical complications. In addition, one-year graft and patient survival do not display inferior results in patients undergoing nighttime transplantation.

## Introduction

Sleep deprivation is a known risk factor for the performance of medical professionals [1]. The adverse effects of sleep deprivation on neurocognitive and psychomotor performance have been demonstrated both in laboratories (using clinically relevant, although artificial tasks) as well as in the setting of real clinical performance [2, 3]. Thus, excessive work hours, circadian disruption and physician fatigue are associated with a higher incidence of preventable medical errors [4]. This is especially important in surgery as the current literature suggests sleep deprivation to be associated with higher surgical complication rates and since the Institute of Medicine identified surgical complications as the second most common cause of preventable morbidity and mortality [5, 6]. To reduce fatigue-related medical adverse events in surgery and thus increase patient’s safety and improve quality of medical care, physicians working hours have been restricted in the European Union as well as in the United States (US) [7–9]. In addition, the general concern regarding a potential risk of overnight procedures has led to a trend towards a critical scrutiny as to which operations can be safely delayed to alter the starting time from night to day [10, 11].

Solid organ transplantation appears to be especially vulnerable since it combines technically challenging operative procedures with an often unpredictable start time, frequently during the night. Accordingly, there are numerous studies analyzing potential adverse effects of nighttime procedures on outcomes after solid organ transplantation [12–17]. However, there are currently only two reports investigating the influence of the operative start time on outcomes after orthotopic liver transplantation (oLT) [18, 19]. In oLT, potential risks of nighttime surgery have to be critically balanced against the key risk factor being cold ischemia time (CIT), which determines outcome of grafts and recipients [20]. Thus, to simply delay the operation start time is not yet feasible. To our best knowledge, outcomes stratified for day versus nighttime oLT have not been investigated in any European transplant center, which display significant differences to American transplant centers in which the before-mentioned studies have been carried out. To address this gap of evidence, we conducted a retrospective single center study in a German transplant center within the Eurotransplant region. The aim of the current study was to analyze whether complications, one-year graft and patient survival correlate with operative start times in oLT.

## Material and methods

### Study design and study population

A total of 350 patients underwent oLT at the department of General, Visceral and Transplant Surgery, University Hospital Muenster, Germany, between January 2006 and December 2017. Follow-up for this study was 12 months. To compare nighttime and daytime surgery, the study population was divided into two groups, based on the start time of oLT. Nighttime procedures were defined as those operations starting between 6 p.m. and 8 a.m., whereas procedures started between 8 a.m. and 6 p.m. were defined as daytime oLT. The respective operative start time was defined by time of skin incision. The study design was a retrospective single center study with a follow-up period of 12 months. Ethical approval for the study was obtained from the local ethics committee (Ethik-Kommission der Ärztekammer Westfalen-Lippe und Westfälischen Wilhelms-Universität, No. 2019-244-f-S). Moreover, all participating patients gave their written consent for routine recording of clinical data. All retrospectively collected data were from patients’ charts, the Eurotransplant Network Information System (ENIS) or in-house transplant data files. Prior to analysis all data were de-identified. The study was conducted in accordance with the ethical principles stated in the Declaration of Helsinki.

### Demographics

Donor parameters (age, gender, body mass index (BMI) and donor center) were extracted from ENIS and a donor risk index (DRI) was calculated accordingly [21]. Recipients age, gender, BMI, model for end-stage liver disease (MELD) score, indication for oLT, high urgency (HU) status, time on waiting list, cold and warm ischemia times as well as numbers of prior transplants were retrospectively collected by electronic record review. The category “other” as indication for oLT includes the following etiologies: amyloidosis, hemochromatosis, autoimmune hepatitis, alpha-1 antitrypsin deficiency, Osler's disease, glycogen storage disease, vanishing bile duct syndrome, traumatic liver rupture, IGG4 associated sclerosing cholangitis, sarcoidosis, angiosarcoma and cryptogenic cirrhosis.

When re-transplantations occurred within one year, they were not counted as additional oLT cases, but were included as a complication of oLT for the respective group of the initial oLT (nighttime or daytime).

### Outcome measures

The primary outcome measures were complications requiring reoperation (grade IIIb according to Clavien-Dindo [22]), one-year patient, death-censored graft and overall graft survival. For the complications an additional categorization was performed as follows: 1) hemorrhage (hematoma or bleeding related to the transplantation), 2) vascular complications (hepatic artery stenosis or thrombosis, portal vein thrombosis and hepatic venous obstruction), 3) complications of the biliary tract (such as stricture, fistula or T-tube dislocation), 4) complication of wound healing (impairment and dehiscence), 5) gastrointestinal causes for complications (ulcer, perforation, bleeding and anastomotic leakage) and 6) other complications. All complications requiring reoperation were further divided into early (<30 day) and late (≥30 days) complications. In addition, wound complications were analyzed for the occurrence of surgical side infections (SSI) within the first 30 days of oLT as defined by the Centers for Disease Control and Prevention (CDC) Hospital Infection Control Practices Advisory Committee [23].

Secondary outcome parameters included 30-day and 90-day patient and graft survival, primary non-function (PNF, defined as graft failure resulting in death or re-transplantation within 30 days of the initial transplant excluding any identifiable cause of graft failure such as rejection or vascular thrombosis). Additional secondary outcome measures were rates of biopsy proven acute rejections (AR), rates of re-transplantations, peak serum values of alanine transaminase (ALT) and aspartate transaminase (AST), length of stay at the intensive care unit (ICU), length of stay in the hospital, frequencies of endoscopic retrograde cholangiopancreatography (ERCP), death within the initial stay as well as the number and length of re-admissions after initial hospital discharge.

### Statistical analysis

Normally distributed continuous variables are presented as mean ± standard deviation (SD) and groups were compared utilizing the student’s t-test. For not normally distributed continuous variables median and interquartile range (IQR, Q_0.25_—Q_0.75_) are given and a comparison between groups was performed with the Mann-Whitney U test. For categorical variables the Fisher's exact test was used.

A logistic regression model was used to estimate the probability of surgical complication based on one or more predictor variables. Included variables were: operative start time (nighttime vs daytime), recipient age, recipient gender, recipient BMI, indication for oLT, cold and warm ischemia time, MELD, time on waiting list, HU status, retransplantation, donor age, gender, BMI, DRI, PNF, biopsy proven rejection, peak AST and ALT, stay at ICU and ERCP. Multivariable model building was performed using a stepwise variable selection procedure (inclusion: *p*-value of the score test ≤ 0.05, exclusion: *p*-value of the likelihood ratio test > 0.1). Significant results are presented as odds ratios (OR) with 95% confidence interval (CI) and *p*-value of likelihood ratio test. For non-selected variables in multivariable analyses, neither p-value of score test nor given OR are reported.

The association between nighttime or daytime oLT and primary outcomes (one-year patient survival, death-censored graft survival and overall graft survival) was tested with Cox proportional hazards regression models with a simultaneous adjustment for potential confounders. Univariate analysis included nighttime or daytime status, recipient age, gender and BMI, indication for oLT, cold and warm ischemia time, MELD, time on waiting list, HU status, prior transplantations, donor age, gender, BMI and donor center as well as PNF, AR, peak of AST and ALT, stay at ICU or hospital, number and length of readmissions, reoperations and re-transplantations, DRI and ERCP. Afterwards, one-year patient survival, death-censored graft survival and overall graft survival were adjusted for PNF, stay at ICU, number and length of readmissions and reoperations, respectively utilizing a stepwise variable selection procedure for covariates with a *p*-value less than 0.05 and a multivariable logistic regression analysis. Significant results are shown as hazard ratios (HR) with 95% CI and *p*-value. For non-selected variables in the multivariable analyses, neither p-value of score test nor given HR are reported. One-year patient survival, death-censored graft and overall graft survival were estimated by Kaplan-Meier methodology and compared using log-rank tests; p-values ≤0.05 were considered statistically significant. All statistical analyses were performed with IBM SPSS Statistics 24 for Windows (IBM Corporation, Somers, NY, USA).

## Results

### Study population characteristics

Three hundred and fifty patients were analyzed for this study and the cohort was subsequently divided into two groups: those who underwent daytime oLT (starting time 8 a.m. to 6 p.m.) and those who underwent nighttime oLT (starting time 6 p.m. to 8 a.m.) While 154 oLTs (44%) were conducted during daytime, 196 (56%) were performed during nighttime, respectively (Table 1).

**Table 1:** Baseline comparison of recipient characteristics in patients with orthotopic liver transplantation (oLT) stratified by the operative starting time (daytime or nighttime).

Recipient characteristics			
	Daytime (n = 154)	Nighttime oLT (n = 196)	*p*-value
**Age** *(mean ± SD)*	51.3 ± 12.1	53.3 ± 11.0	0.115^a^
**Gender** *(% males)*	63.0	65.3	0.655^b^
**BMI** *(kg/m*^*2*^, *median (Q*_*0*.*25*_, *Q*_*0*.*75*_*))*	25.7 (22.8, 29.4)	25.4 (22.9, 29.5)	0.876^c^
**Indications for transplant** *(%)*			0.636^e^
ALF	12.3	13.2	
HCC	20.8	25.5	
Viral Hepatitis	14.3	12.8	
PSC, PBC, SSC	10.4	10.7	
Alcoholic Cirrhosis	14.3	18.4	
PLD	4.5	2.0	
Other	23.4	17.4	
**Cold ischaemia time** *(h*, *mean ± SD)*	10.1 ± 2.6	10.0 ± 2.6	0.798^a^
**Warm ischaemia time** *(min*, *mean ± SD)*	40.6 ± 9.3	41.2 ± 9.4	0.580^a^
**MELD** *(mean ± SD)*	22.1 ± 12.4	22.3 ± 12.2	0.928^a^
**Time on waiting list** *(d*, *median (Q*_*0*.*25*_, *Q*_*0*.*75*_*))*	126.5 (21.8, 355.5)	103.0 (16.0, 319.5)	0.367^c^
**HU Status** *(% HU)*	4.5	5.1	0.807^b^
**≥ 1 prior transplant** *(number*, *%)*	17 (11.0)	10 (5.1)	0.045^b^

Data are presented as mean ± standard deviation (SD), median, interquartile range (Q_0.25_—Q_0.75_) or relative frequencies. Categorical variables were compared using Fisher's exact test while continuous variables were compared using Student’s *t*-test (normally distributed) or Mann-Whitney U test (not normally distributed). BMI = body mass index, ALF = acute liver failure, HCC = hepatocellular carcinoma, PSC = primary sclerosing cholangitis, PBC = primary biliary cholangitis, SSC = secondary sclerosing cholangitis, PLD = polycystic liver disease, MELD = model for end-stage liver disease, HU = high urgency.

^a)^ Student’s *t*-test,

^b)^ Fisher's exact test,

^c)^ Mann-Whitney U test,

a *p*-value less than 0.05 was considered statistically significant.

Recipient characteristics showed patient age, gender and BMI to be comparable between daytime and nighttime oLT groups, while significantly more patients in the daytime group (11% vs. 5.1%, *p* = 0.045) had received a prior transplant (Table 1). Hepatocellular carcinoma represented the most common indication for oLT in both daytime (20.8%) and nighttime (25.5%) groups, followed by viral hepatitis (14.3%) in the daytime group and alcoholic cirrhosis (18.4%) in the nighttime group. The mean MELD score showed no differences between daytime (22.1) and nighttime (22.3) transplantation. Similarly, time on waiting list was comparable between the two groups (126.5 vs. 103.0 days). The start time of oLT had no influence on cold (daytime 10.1 ± 2.6 hours; nighttime 10.0 ± 2.6 hours) or warm (daytime 40.6 ± 9.3 minutes; nighttime 41.2 ± 9.4 minutes) ischemia times (Table 1).

As for donor characteristics, donors in the daytime group were significantly younger (daytime 49.4 ± 14.7 years; nighttime 54.5 ± 14.7 years; *p* = 0.001) and showed a significantly lower DRI (daytime 1.728 ± 0.3; nighttime 1.820 ± 0.3; *p* = 0.016) (Table 2). Donor gender and donor BMI were comparable. For daytime oLT, 88.3% and 86.7% for nighttime oLT respectively, of the organs were procured at a national donor center with no significant differences between the two groups (Table 2).

**Table 2:** Baseline donor characteristics stratified by nighttime or daytime orthotopic liver transplantation.

Donor characteristics			
	Daytime (n = 154)	Nighttime oLT (n = 196)	*p*-value
**Age** *(mean ± SD)*	49.4 ± 14.7	54.5 ± 14.7	0.001^a^
**Gender** *(% males)*	53.9	57.1	0.588^b^
**BMI** *(kg/m*^*2*^, *median (Q*_*0*.*25*_, *Q*_*0*.*75*_*))*	25.0 (23.7, 27.7)	25.7 (23.7, 28.9)	0.262^c^
**Donor Center** (*% national*)	88.3	86.7	0.746^b^
**DRI** (*mean ± SD*)	1.728 ± 0.368	1.820 ± 0.340	0.016^a^

Results are presented as mean ± standard deviation (SD), median, interquartile range (Q_0.25_—Q_0.75_) or relative frequencies. Categorical variables were compared using Fisher's exact test while continuous variables were compared using Student’s *t*-test (normally distributed) or Mann-Whitney U test (not normally distributed). BMI = body mass index, DRI = donor risk index.

^a)^ Student’s *t*-test,

^b)^ Fisher's exact test,

^c)^ Mann-Whitney U test,

a *p*-value less than 0.05 was considered statistically significant.

### Surgical complications

**Fig 1:**
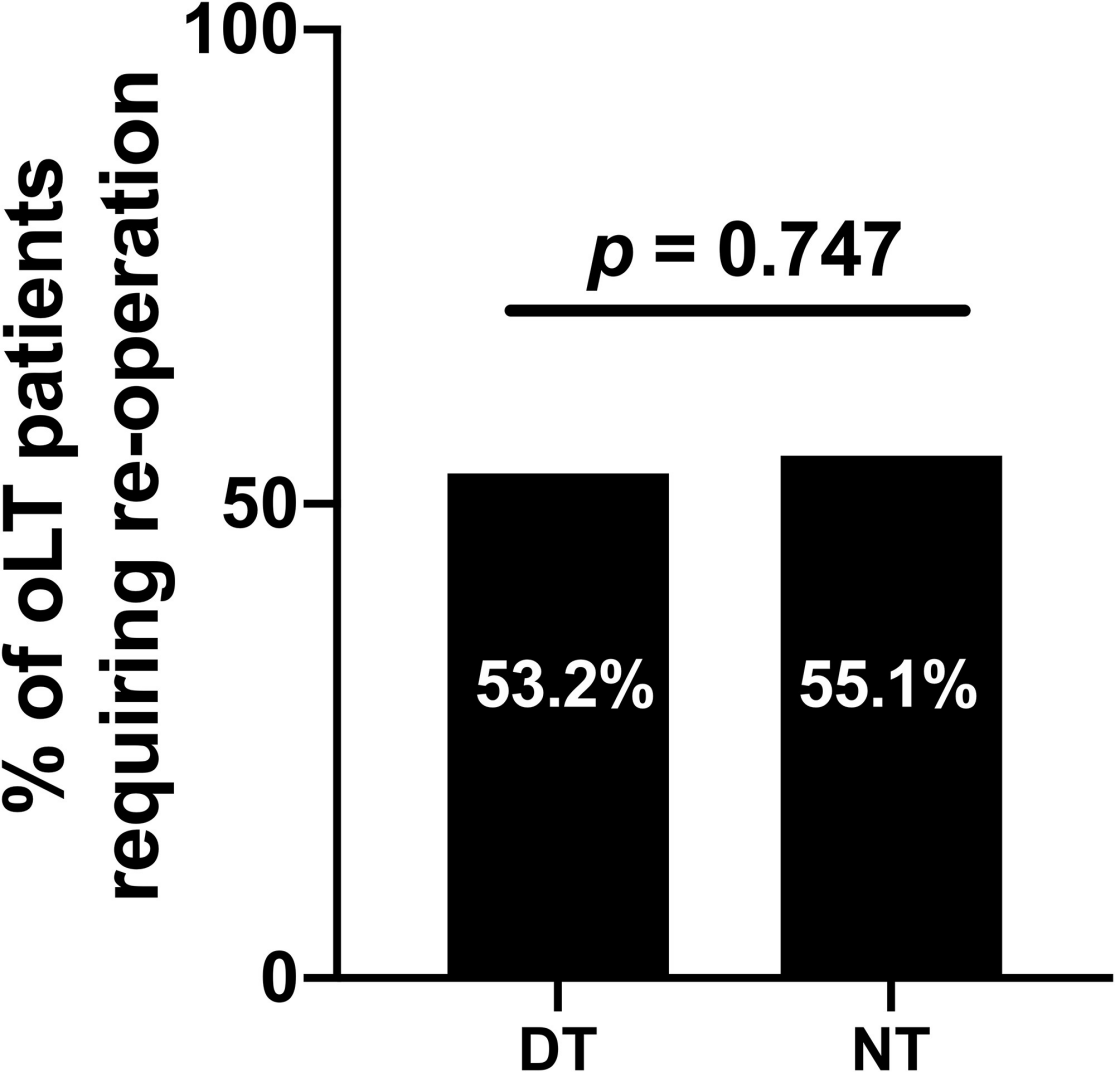
Frequency of surgical complications requiring reoperation. Groups were stratified for daytime (DT) and nighttime (NT) orthotopic liver transplant (oLT) recipients.

Frequencies of surgical complications requiring reoperation were comparable between daytime (53.2%) and nighttime (55.1%, p = 0.747) and oLT (Fig 1). The same accounted for average and maximal numbers of reoperation among these patients. When analyzed for the respective indication for reoperation, haemorrhage was the leading reason for an operative revision in both groups. (Table 3). Surgical complications were further divided into early (<30 day) and late (≥30 days) complications and 85% of all complications occurred during the early postoperative phase, with no differences in frequencies for daytime (87.8%) and nighttime (83.3%) procedures (Table 3). The most common indication within the early group were again bleeding complications, with no differences (p = 0.957) between the daytime (42.5%) and nighttime (43.3%) group. Among all early wound complications (15.1% for daytime and 11.1% for nighttime oLT), 50% meet the CDC criteria for SSI II-III, with the other ones being non-infectious wound complications. When frequencies of SSIs were compared, no differences were for rates of SSI II-III between daytime and nighttime patients. In summary, operative start time of oLT (nighttime vs. daytime) was not associated with the occurrence of surgical complications requiring reoperation in the univariable analyses nor in a multivariable analysis adjusted for potential confounders (OR 0.928 (95% CI 0.608–1.417), *p* = 0.729) (Table 4).

**Table 3:** Frequencies and indications of surgical complications requiring re-operation following orthotopic liver transplantation (oLT) stratified by operative starting time (nighttime or daytime).

Results			
	Daytime (n = 154)	Nighttime oLT (n = 196)	*p*-value
**Reoperation** *(%)*	53.2	55.1	0.747^b^
**Time of reoperation** *(%)*			0.417^b^
Early (<30d)	87.8	83.3	
Late (>30d)	12.2	16.7	
**Indications for reoperation** *(%)*			
Haemorrhage	37.8	38.0	0.856^c^
Vascular complications	3.7	7.4	0.257^c^
Biliary tract complications	8.5	11.1	0.519^c^
Wound complications	17.1	12.0	0.393^c^
Gastrointestinal complications	0	1.9	0.209^c^
Other	32.9	29.6	0.765^c^
**Number of reoperations** *(median MIN*, *MAX)*	1.0 (1, 14)	1.0 (1, 12)	0.618^c^

Results are presented as median (with minimal and maximal values) or relative frequencies. Categorical variables were compared using Fisher's exact test while continuous variables were compared using Student’s *t*-test (normally distributed). PNF = primary non-function, AST = aspartate aminotransferase, ALT = alanine aminotransferase, ICU = intensive care unit.

^a)^ Student’s *t*-test,

^b)^ Fisher's exact test,

a *p*-value less than 0.05 was considered statistically significant.

**Table 4:** Logistic regression model for predictors of surgical complications.

Logistic regression model for predictors of surgical complications		
Parameters	Univariate OR (95% CI) *p*-value	Multivariate OR (95% CI) *p*-value
**Operative start time** (nighttime vs daytime)	0.928 (0.608–1.417) 0.729	
**Recipient age** (years)	0.993 (0.975–1.011) 0.447	
**Recipient gender** (male vs female)	1.114 (0.717–1.729) 0.631	
**Recipient BMI** (kg/m^2^)	1.016 (0.976–1.057) 0.434	
**Indication for oLT**	1.252 (0.763–2.055) 0.374	
**Cold ischemia time** (hours)	0.952 (0.878–1.032) 0.232	
**Warm ischemia time** (minutes)	1.041 (1.017–1.066) 0.001	1.059 (1.027–1.092) < 0.001
**MELD**	1.031 (1.013–1.051) 0.001	
**Time on waiting list** (days)	1.000 (0.999–1.000) 0.382	
**HU status** (yes vs no)	0.478 (0.165–1.388) 0.175	
**≥ 1 prior transplant** (yes vs no)	0.314 (0.123–0.797) 0.015	0.327 (0.117–0.909) 0.032
**Donor age** (years)	1.013 (0.999–1.027) 0.078	
**Donor gender** (male vs female)	1.198 (0.784–1.830) 0.405	
**Donor BMI** (kg/m^2^)	1.004 (0.958–1.052) 0.881	
**DRI**	1.696 (0.924–3.113) 0.088	
**PNF** (yes vs no)	0.073 (0.017–0.313) < 0.001	0.050 (0.006–0.401) 0.005
**Biopsy proven rejection** (yes vs no)	0.818 (0.452–1.478) 0.505	
**Peak AST** (U/l)	1.000 (1.000–1.000) 0.022	
**Peak ALT**(U/l)	1.000 (1.000–1.000) 0.025	1.000 (1.000–1.000) 0.029
**Stay at ICU** (days)	1.034 (1.019–1.050) < 0.001	1.035 (1.018–1.052) < 0.001
**ERCP** (yes vs. no)	0.713 (0.457–1.110) 0.134	

OR = odds ratios, CI = 95% confidence interval. BMI = body mass index, MELD = model for end-stage liver disease, HU = high urgency, PNF = primary non-function, AST = aspartate aminotransferase, ALT = alanine aminotransferase, ICU = intensive care unit, DRI = donor risk index, oLT = orthotopic liver transplantation, ERCP = endoscopic retrograde cholangiopancreatography

### One-year patient and graft survival

**Fig 2:**
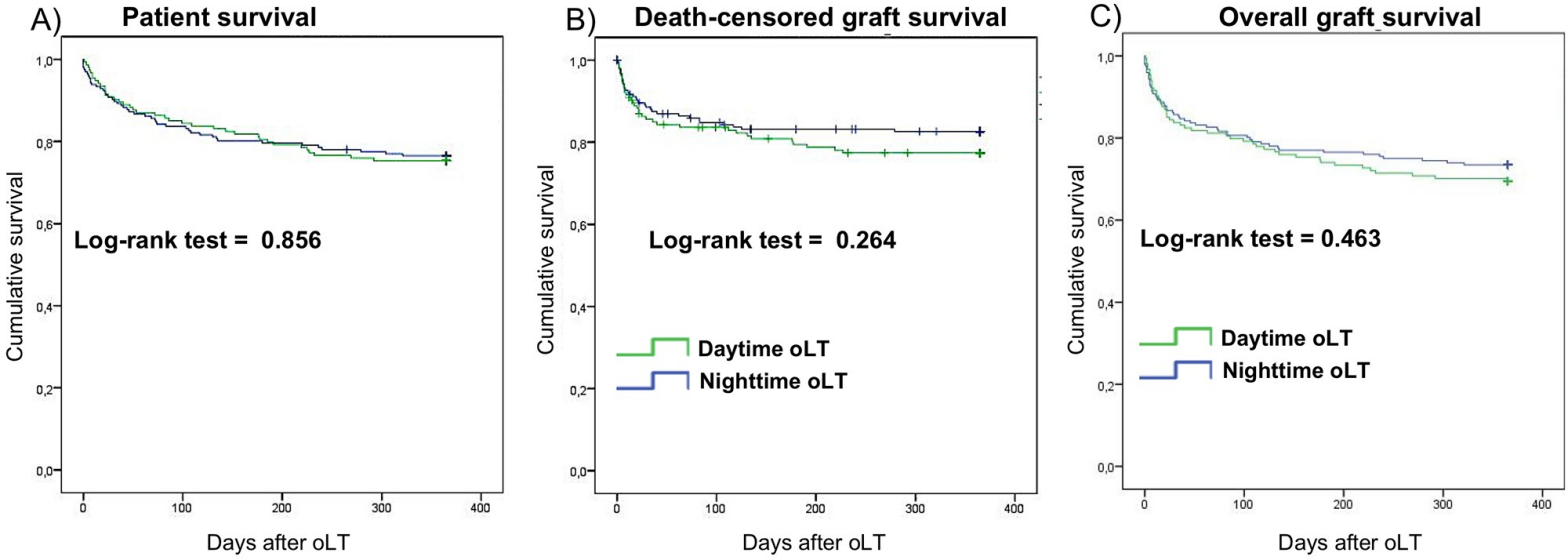
Kaplan-Meier curves for one-year patient and graft survival. Longitudinal survivals of patient survival (**A**), death-censored graft survival (**B**) and overall graft survival (**C**) stratified for daytime and nighttime orthotopic liver transplantation (oLT). Survival rates of daytime (green lines) and nighttime (blue lines) oLT recipients were estimated by Kaplan-Meier methodology and compared by log-rank test.

Kaplan-Meier analysis was used to generate survival curves for one-year patient (Fig 2A), death-censored graft (Fig 2B) and overall graft survival (Fig 2C) for daytime and nighttime oLT. Comparison of patient survival on day 30 (daytime 90.9%; nighttime 90.3%), day 90 (daytime 85.1%; nighttime 82.2%) and after one year (daytime 75.3%; nighttime 76.5%) revealed no significant differences between groups. Similarly, graft survival on day 30 (daytime 83.1%; nighttime 85.7%), day 90 (daytime 79.2%; nighttime 80.1%) and after one year (daytime 69.5%; nighttime 73.5%) were comparable. Occurrence of death within the first 30 days following oLT (daytime 9.1%; nighttime 9.7%) and death within the initial hospital stay (daytime 16.2%; nighttime 18.4%) were not different between groups (Table 5). For the unadjusted Cox proportional hazard modeling we could show that nighttime patients had a 1.040 (0.677–1.599 95% CI) hazard of death at 356 days (Table 6), a 1.159 (0.781–1.719 95% CI) hazard of overall graft loss (Table 7) and a 1.312 (0.813–2.118 95% CI) hazard of death censored graft loss (Table 8).

**Table 5:** Primary and secondary outcomes for orthotopic liver transplantation (oLT) stratified by operative starting time (nighttime or daytime).

Results			
	Daytime (n = 154)	Nighttime oLT (n = 196)	*p*-value
**Patient survival** *(%)*			
30 d	90.9	90.3	0.823^d^
90 d	85.1	83.2	0.623^d^
1 y	75.3	76.5	0.856^d^
**Graft survival** *(%)*			
30 d	83.1	85.7	0.555^d^
90 d	79.2	80.1	0.862^d^
1 y	69.5	73.5	0.463^d^
**Re-oLT within 1y** *(%)*	12.3	7.1	0.139^b^
**PNF** *(%)*	9.7	7.7	0.565^b^
**Biopsy proven Rejection** *(%)*	15.6	14.8	0.881^b^
**Peak AST** *(U/l*, *median (Q*_*0*.*25*_, *Q*_*0*.*75*_*))*	3628.5 (1663.3, 8882.5)	3095.0 (1380.0, 8010.0)	0.242^c^
**Peak ALT** *(U/l*, *median (Q*_*0*.*25*_, *Q*_*0*.*75*_*))*	2081.5 (954.0, 4573.5)	1654.0 (742.0, 4395.0)	0.334^c^
**Stay at ICU** *(d*, *median (Q*_*0*.*25*_, *Q*_*0*.*75*_*))*	7.0 (3.0, 24.0)	7.0 (3.0, 22.0)	0.673^c^
**Initial hospital stay** *(d*, *median (Q*_*0*.*25*_, *Q*_*0*.*75*_*))*	36.0 (20.8, 70.0)	34.5 (21.0, 57.8)	0.533^c^
**Death within 30d** *(%)*	9.1	9.7	1.000^b^
**Death within initial stay** *(%)*	16.2	18.4	0.671^b^
**Number of readmissions** *(mean ± SD)*	1.8 ± 1.9	2.0 ± 2.1	0.500^a^
**Length of readmissions** *(d*, *median (MIN*, *MAX))*	23.0 (1.132)	22.0 (1.215)	0.798^c^
**ERCP** *(%)*	31.8	38.3	0.218^b^

Results are presented as mean ± standard deviation (SD), median, interquartile range (Q_0.25_—Q_0.75_) or relative frequencies. Categorical variables were compared using Fisher's exact test while continuous variables were compared using Student’s *t*-test (normally distributed) or Mann-Whitney U test (not normally distributed). PNF = primary non-function, AST = aspartate aminotransferase, ALT = alanine aminotransferase, ICU = intensive care unit, ERCP = endoscopic retrograde cholangiopancreatography

^a)^ Student’s *t*-test,

^b)^ Fisher's exact test,

^c)^ Mann-Whitney U test and

^d)^ Log-rank test,

a *p*-value less than 0.05 was considered statistically significant.

**Table 6:** Cox proportional hazards regression model with univariate and multivariable logistic regression analyses of one-year patient survival.

Cox regression model for 1-year patient survival		
Parameters	Univariate HR (95% CI) *p*-value	Multivariate HR (95% CI) *p*-value
**Operative start time** (nighttime vs daytime)	1.040 (0.677–1.599) 0.856	
**Recipient age** (years)	1.012 (0.992–1.032) 0.243	
**Recipient gender** (male vs female)	0.941 (0.600–1.475) 0.790	
**Recipient BMI** (kg/m^2^)	0.987 (0.947–1.029) 0.547	
**Indication for oLT**	0.893 (0.549–1.452) 0.648	
**Cold ischemia time** (hours)	0.989 (0.909–1.075) 0.789	
**Warm ischemia time** (minutes)	1.006 (0.983–1.030) 0.593	
**MELD**	1.035 (1.016–1.055) < 0.001	
**Time on waiting list** (days)	1.000 (0.999–1.000) 0.234	
**HU status** (yes vs no)	0.536 (0.234–1.230) 0.141	
**≥ 1 prior transplant** (yes vs no)	0.617 (0.309–1.232) 0.171	
**Donor age** (years)	1.010 (0.995–1.025) 0.188	
**Donor gender** (male vs female)	1.262 (0.823–1.936) 0.286	
**Donor BMI** (kg/m^2^)	0.987 (0.939–1.036) 0.592	
**Donor center** (national vs international)	0.731 (0.353–1.515) 0.400	
**DRI**	1.209 (0.662–2.207) 0.537	
**Re-oLT within 1y** (yes vs no)	0.276 (0.165–0.461) < 0.001	
**PNF** (yes vs no)	0.124 (0.076–0.204) < 0.001	0.434 (0.241–0.783) 0.006
**Biopsy proven rejection** (yes vs no)	0.822 (0.470–1.436) 0.491	
**Peak AST** (U/l)	1.000 (1.000–1.000) 0.217	
**Peak ALT**(U/l)	1.000 (1.000–1.000) 0.950	
**Stay at ICU** (days)	1.003 (1.001–1.005) 0.015	
**Initial hospital stay** (days)	1.001 (0.999–1.004) 0.299	
**Number of readmissions**	0.385 (0.294–0.504) < 0.001	0.403 (0.303–0.536) < 0.001
**Length of readmissions** (days)	1.009 (0.998–1.020) 0.102	
**Reoperation** (yes vs no)	0.237 (0.138–0.409) < 0.001	0.247 (0.130–0.469) < 0.001
**Number of reoperations**	1.242 (1.172–1.317) < 0.001	
**ERCP** (yes vs. no)	1.228 (0.777–1.941) 0.380	

HR = hazard ratios, CI = 95% confidence interval. MELD = model for end-stage liver disease, BMI = body mass index, PNF = primary non-function, HU = high urgency, Tx = transplantation, AST = aspartate aminotransferase, ALT = alanine aminotransferase, ICU = intensive care unit, oLT = orthotopic liver transplantation, ERCP = endoscopic retrograde cholangiopancreatography

**Table 7:** Cox proportional hazards regression model with univariate and multivariable logistic regression analyses of one-year overall graft survival.

Cox regression model for 1-year overall graft survival		
Parameters	Univariate HR (95% CI) *p*-value	Multivariate HR (95% CI) *p*-value
**Operative start time** (nighttime vs daytime)	1.159 (0.781–1.719) 0.465	
**Recipient age** (years)	1.000 (0.983–1.018) 0.984	
**Recipient gender** (male vs female)	1.099 (0.731–1.651) 0.650	
**Recipient BMI** (kg/m^2^)	0.986 (0.949–1.024) 0.463	
**Indication for oLT**	0.887 (0.567–1.388) 0.601	
**Cold ischemia time** (hours)	0.988 (0.914–1.067) 0.756	
**Warm ischemia time** (minutes)	0.997 (0.976–1.019) 0.803	
**MELD**	1.033 (1.015–1.051) < 0.001	
**Time on waiting list** (days)	1.000 (0.999–1.000) 0.567	
**HU status** (yes vs no)	0.426 (0.207–0.879) 0.021	
**≥ 1 prior transplant** (yes vs no)	0.591 (0.316–1.107) 0.100	
**Donor age** (years)	1.006 (0.992–1.019) 0.418	
**Donor gender** (male vs female)	1.516 (1.022–2.250) 0.039	
**Donor BMI** (kg/m^2^)	0.973 (0.928–1.020) 0.252	
**Donor center** (national vs international)	0.769 (0.400–1.479) 0.432	
**DRI**	1.177 (0.673–2.057) 0.568	
**PNF** (yes vs no)	0.027 (0.016–0.049) < 0.001	0.116 (0.060–0.227) < 0.001
**Biopsy proven rejection** (yes vs no)	0.793 (0.476–1.322) 0.374	
**Peak AST** (U/l)	1.000 (1.000–1.000) 0.044	
**Peak ALT**(U/l)	1.000 (1.000–1.000) 0.383	
**Stay at ICU** (days)	1.003 (1.001–1.005) 0.005	
**Initial hospital stay** (days)	1.002 (1.000–1.004) 0.100	
**Number of readmissions**	0.495 (0.406–0.603) < 0.001	0.574 (0.465–0.708) < 0.001
**Length of readmissions** (days)	1.008 (0.998–1.017) 0.105	
**Reoperation** (yes vs no)	0.211 (0.127–0.353) < 0.001	0.285 (0.149–0.544) < 0.001
**Number of reoperations**	1.355 (1.277–1.438) < 0.001	1.081 (1.000–1.168) 0.049
**ERCP** (yes vs. no)	1.289 (0.843–1.971) 0.242	

HR = hazard ratios, CI = 95% confidence interval. MELD = model for end-stage liver disease, BMI = body mass index, PNF = primary non-function, HU = high urgency, Tx = transplantation, AST = aspartate aminotransferase, ALT = alanine aminotransferase, ICU = intensive care unit, oLT = orthotopic liver transplantation, ERCP = endoscopic retrograde cholangiopancreatography

**Table 8:** Cox proportional hazards regression model with univariate and multivariable logistic regression analyses of one-year death-censored graft survival.

Cox regression model for 1-year death-censored graft survival		
Parameters	Univariate HR (95% CI) *p*-value	Multivariate HR (95% CI) *p*-value
**Operative start time** (nighttime vs daytime)	1.312 (0.813–2.118) 0.266	
**Recipient age** (years)	0.999 (0.978–1.020) 0.930	
**Recipient gender** (male vs female)	0.960 (0.580–1.589) 0.873	
**Recipient BMI** (kg/m^2^)	1.004 (0.960–1.049) 0.870	
**Indication for oLT**	1.014 (0.579–1.779) 0.960	
**Cold ischemia time** (hours)	0.959 (0.873–1.054) 0.389	
**Warm ischemia time** (minutes)	1.000 (0.974–1.027) 0.993	
**MELD**	1.036 (1.014–1.059) 0.001	
**Time on waiting list** (days)	1.000 (0.999–1.000) 0.562	
**HU status** (yes vs no)	0.281 (0.134–0.588) 0.001	0.404 (0.159–1.030) 0.058
**≥ 1 prior transplant** (yes vs no)	0.559 (0.267–1.171) 0.123	
**Donor age** (years)	1.004 (0.988–1.021) 0.625	
**Donor gender** (male vs female)	1.577 (0.976–2.550) 0.063	
**Donor BMI** (kg/m^2^)	0.945 (0.887–1.006) 0.074	
**Donor center** (national vs international)	0.937 (0.448–1.960) 0.862	
**DRI**	1.256 (0.636–2.483) 0.512	
**PNF** (yes vs no)	0.016 (0.009–0.031) < 0.001	0.064 (0.031–0.130) < 0.001
**Biopsy proven rejection** (yes vs no)	1.017 (0.519–1.992) 0.961	
**Peak AST** (U/l)	1.000 (1.000–1.000) 0.009	
**Peak ALT**(U/l)	1.000 (1.000–1.000) 0.123	
**Stay at ICU** (days)	1.003 (1.001–1.006) 0.010	
**Initial hospital stay** (days)	1.002 (1.000–1.005) 0.085	
**Number of readmissions**	0.418 (0.315–0.556) < 0.001	0.560 (0.422–0.743) < 0.001
**Length of readmissions** (days)	1.006 (0.993–1.020) 0.370	
**Reoperation** (yes vs no)	0.115 (0.053–0.253) < 0.001	0.153 (0.059–0.398) < 0.001
**Number of reoperations**	1.388 (1.298–1.484) < 0.001	
**ERCP** (yes vs. no)	1.127 (0.680–1.866) 0.643	

HR = hazard ratios, CI = 95% confidence interval, MELD = model for end-stage liver disease, BMI = body mass index, PNF = primary non-function, HU = high urgency, AST = aspartate aminotransferase, ALT = alanine aminotransferase, ICU = intensive care unit, oLT = orthotopic liver transplantation, ERCP = endoscopic retrograde cholangiopancreatography

### Secondary outcome measures

Frequencies of re-transplantations within the first year showed no significant differences (daytime 12.3%, nighttime 7.1%, p = 0.139). Similarly, frequencies of PNF (daytime 9.7%; nighttime 7.7%, *p* = 0.565) and episodes of biopsy proven rejection (daytime 15.6%; nighttime 18.8%, *p* = 0.881) were similar between daytime and nighttime groups (Table 5). In addition, peak values of liver-specific enzymes (AST, ALT) were comparable between daytime and nighttime transplantation groups. Both median length of stay at ICU (daytime 7.0 days; nighttime 7.0 days) and initial hospital stay (daytime 36.0 days; nighttime 34.5 days) exhibited no significant differences. The length of stay following readmission was the same in both groups (daytime 23.0 days; nighttime 22.0 days) (Table 5).

## Discussion

To the best of our knowledge this study provides the first analyses of outcomes of liver transplant recipients stratified for daytime or nighttime surgery within the Eurotransplant region. We found no differences in postoperative complications requiring re-operation as well as no differences in one year-patient and one year-graft survival between daytime and nighttime transplantation. Our results are encouraging for patients as well as for the transplant community that the implemented safety instruments and quality measures can ensure liver transplantation as a safe, standardized and reliable procedure, irrespective of the operative starting time.

Liver transplantation is a technically highly challenging procedure and its success depends to a great extent on the surgical performance, especially since the continuous advancement in immunosuppressive therapies have further reduced the incidence of non-surgery-related causes of graft loss. Although any form of medical errors is multifactorial, compelling evidence suggests that sleep deprivation and fatigue are significant risk factors for surgical complications. Possible short-term consequences of fatigue-related complications might include early re-operations due to surgical related errors such as hemorrhage or vascular complications, while later complications could involve the biliary tract or wound healing disorders. In our cohort, nighttime oLT was not associated with a higher frequency of surgical complications requiring reoperation when compared to daytime oLT. In contrast to our results, an association between sleep deprivation and reduced surgical performance has been reported. Grantcharov et al. as well as Taffinder et al. used models with laparoscopic simulation to demonstrate higher rates of errors in the performance of surgeons on post-call mornings [24, 25]. In addition, Ricci et al. analyzed orthopedic surgery and found that nighttime operations were associated with higher frequencies of reoperation [26]. In the field of colorectal surgery, Komen et al. revealed that anastomotic leakage after colorectal surgery showed a twofold increase in a cohort of nighttime surgery, with the operative start time being an independent risk factor [27]. Thus, we can only speculate why our data reveal no differences in complications rates between daytime and nighttime transplantation. One reason could be that we use highly specialized transplant teams, with a high caseload per surgeon and a high caseload at our center with a standardized operative procedure. In addition, sleep deprivation and fatigue not only apply to surgeons but also to the anesthesia staff, scrub nurses, as well as to intensive care unit staff who manage the patient in the early post-operative phase. Unlike other nighttime emergency procedures, in oLT, the intraoperative anesthesia as well as the postoperative care are more likely being conducted by a board-certified senior anesthesiologist and not by residents. Thus, the seniority of the involved physicians (surgeons as well as anesthesiologists) might counteract possible negative effects of sleep deprivation. Studies in renal transplantation suggested a difference in the leading surgeon to be a bias when analyzing effects of nighttime operations [12, 16]. Daytime surgery might be used to train inexperienced residents and fellows, while nighttime surgery could be performed without a transplant consultant present. However, in our center all oLTs were conducted by a small group of highly experienced consultant transplant surgeons, irrespective of the starting time. Thus, we would exclude a reduced experience during the night as a potential bias.

In the field of oLT, there are currently two studies addressing a possible effect of nighttime surgery on outcomes in liver transplantation. Orman et al. analyzed data from the United Network for Organ Sharing (UNOS) database, combining a total of 94,768 liver transplants from 150 US centers. They found no differences in patient and graft survival rates at 30-, 90- and 365 days when patients were stratified for nighttime and daytime procedures [19]. When comparing our results with the reported graft- and patient survival rates from Orman et al. one has to consider important differences between the studies. First of all, Orman reports a retrospective UNOS database analysis including data from 1987 to 2010, while we report a single center experience during a fairly narrow timeframe of eleven years. In addition, there are significant differences in DRI, allocation and MELD score at oLT between the UNOS and ET region [28]. In a second US study, Lonze et al. analyzed 587 adult liver transplant recipients and found nighttime transplantations to be slightly longer in duration and to be associated with a higher use of blood products compared to daytime procedures. In addition, they revealed a two-fold greater risk of early (within 7 days of transplant) postoperative death in the nighttime group [18]. Concerning other solid organ transplantation with respect to nighttime procedures, the most sophisticated study on the topic of nighttime procedures in kidney transplantation was reported by the group of Schrem et al. who created a risk balancing score and suggested to avoid kidney transplantation between 3 a.m and 6 a.m. as long as the CIT can be limited to 23.5 hours [17]. In line with this, Fechner et al. analyzed a cohort of 260 kidney transplantation patients and found a higher risk for re-operation (especially for vascular complications) following nighttime procedures. In addition, nighttime transplantation was associated with a higher risk of graft failure [14]. In contrary to these findings Shaw et al., report a decrease in vascular complications in a nighttime cohort of kidney transplant recipients and no differences in patient or graft survival between day- and nighttime procedures [13]. This was supported by data from Austria from Kienzl-Wagner et al., who stratified 873 deceased donor kidney transplants for day- or nighttime operations. They as well did not observe differences in the frequencies of complications and no differences in patient or graft survival rates [12]. Seow et al. state that in their cohort of 322 adult kidney transplant recipients, the incidence of complications was unaffected by the starting time of surgery [29]. Van Brunschot et al. even reported beneficial effects of nighttime procedures on pure technical graft failure rates when analyzing 4,519 renal transplantations from the Dutch Organ Transplant Registry [16].

Beyond the described comparable results for short-term outcomes in our cohort (such as surgical-related complications requiring reoperation), nighttime oLT was also not associated with higher frequencies of re-transplantations, PNF or biopsy proven rejection. Short term survival at 30-days and 90-days as well as one-year patient and graft survival was not different between groups. When analyzing donor characteristics, we found age and DRI among donors for the nighttime cohort to be noticeably different. A possible explanation for this finding is that these organs were classified as marginal organs and thus maximal efforts were made to keep the CIT as short as possible by enforcing operative start times during the night. In general, a constant risk balancing between CIT and a potential influence of a nighttime effect is the base of daily decision making in transplant centers all around the world. It is undoubtable that the reduction of CIT is one of the undisputed dogmas of transplant medicine. Prolonging CIT would not only negatively affect outcomes following oLT but would probably increase discard rates of marginal organs [30, 31]. Thus, delaying the recipient operations does not seem favorable in oLT. Since the starting time of oLT is mainly determined by donor organ availability and time of organ procurement, one might suggest to delay the organ procurement. However, this could result in a further damage of the procured organs due to prolonged brain death associated effects and would further increase financial and organizational burdens for donor hospitals [32, 33].

Our data provide first evidence from the Eurotransplant region for an absent negative effect of nighttime procedures on outcomes following oLT. This further testifies the established safety measures that are in place in our transplant center. Based on the results presented here, oLT should inevitably be performed at any time of day in order to minimize CIT and thus to maximize transplant outcomes. Although we could not show an association between nighttime procedures and inferior outcomes, it appears to be logical that rested personal is desirable. Therefore, one has to consider additional steps which could be undertaken to further eliminate any possible negative effect of nighttime procedures in transplant medicine. Among the most promising options is machine perfusion, a tool to possibly increase storage times of organs above the limit of current static cold storage. While the extension of CIT by the use of machine perfusion and hence delaying the operation starting time might be possible in kidney transplantation, it appears to be currently unrealistic as a general alternative in oLT, although promising progress has been made with normothermic continuous machine perfusion for up to 24h [34, 35].

It is important to recognize certain limitations in the study we present here. First of all, the definition of daytime and nighttime surgery is always arbitrary with a potential misclassification bias. We chose a time stratification scheme defining daytime procedures as those between 8 a.m. to 6 p.m. and the nighttime procedures as those from 6 p.m. to 8 a.m. since this classification most reliably represents the regular working hours in our hospital (7 a.m. to 6 p.m). In addition, it appears to be most likely that early morning operation (e.g 06:30 a.m.) are still be done by the nightshift team). Furthermore, the grouping in nearly equal ten- and fourteen-hour blocks enabled us to have sufficient patient numbers for multivariate statistical analyses. However, even when other time strata were applied (daytime procedures: 6 a.m. to 6 p.m. and nighttime procedures:6 p.m. to 6 a.m) the results remained consistent. An additional limitation is the missing information about organ procurement times. Orman et al. have shown that daytime oLT was more likely to involve livers which were procured at night, and as such this could be an additional bias in our retrospective study [19]. It would also be informative to further compare the exact volume of blood loss during bleeding related reoperations as well as intra- or postoperatively transfused packed red blood cells. Another factor that has to be considered when analyzing the data is that other worktime related factors (such as weekends, holiday) can be a potential bias. Our group has recently described weekend surgery to be a risk factor for surgical complications following renal transplantation [36] while it had no negative influence on one-year outcome in our oLT cohort [37].

In summary, our retrospective single center data derived from a German transplant center within the Eurotransplant region provides evidence that oLT is a safe procedure irrespective of the starting time. Our data provides evidence that compared to daytime surgery nighttime liver transplantation is not associated with a greater risk of surgical complications. In addition, one-year graft and patient survival do not display inferior results in patients undergoing nighttime transplantation.
